# Variable Filtered-Waveform Variational Mode Decomposition and Its Application in Rolling Bearing Fault Feature Extraction

**DOI:** 10.3390/e27030277

**Published:** 2025-03-07

**Authors:** Nuo Li, Hang Wang

**Affiliations:** 1 Key Subject Laboratory of Nuclear Safety and Simulation Technology, College of Nuclear Science and Technology, Harbin Engineering University, Harbin 150001, China; linuo2314@163.com; 2 Nuclear Power Institute of China, Chengdu 610213, China

**Keywords:** variational mode decomposition, wideband signal, mode mixing, Wiener filter, bearing fault diagnosis, envelope spectral entropy

## Abstract

Variational Mode Decomposition (VMD) serves as an effective method for simultaneously decomposing signals into a series of narrowband components. However, its theoretical foundation, the classical Wiener filter, exhibits limited adaptability when applied to broadband signals. This paper proposes a novel Variable Filtered-Waveform Variational Mode Decomposition (VFW-VMD) method to address critical limitations in VMD, particularly in handling broadband and chirp signals. By incorporating fractional-order constraints and dynamically adjusting filter waveforms, the proposed algorithm effectively mitigates mode mixing and over-smoothing issues. The mathematical framework of VFW-VMD is formulated, and its decomposition performance is validated through simulations involving both synthetic and real-world signals. The results demonstrate that VFW-VMD exhibits superior adaptability in extracting broadband signals and effectively captures more rolling bearing fault features. This work advances signal processing techniques, enhancing capability and significantly improving the performance of practical bearing fault diagnostic applications.

## 1. Introduction

Rolling bearings represent a cornerstone in modern mechanical systems, serving as indispensable components across a diverse array of applications, including rotating machinery, wind turbines, and automotive systems [1,2,3]. Their primary function is to support rotating shafts, minimize friction, and sustain both radial and axial loads, thereby ensuring the operational stability and reliability of mechanical systems [4]. Given the high-load conditions and complex operational environments in which rolling bearings function, they are prone to degradation mechanisms such as wear, fatigue, and insufficient lubrication. These issues, if left unaddressed, can precipitate severe mechanical failures, potentially leading to costly downtime and safety hazards. Consequently, effective fault feature extraction of rolling bearings is imperative to maintain equipment integrity, enhance operational efficiency, and extend service life [5].

Feature extraction is a critical aspect of rolling bearing fault diagnosis, as it enables the identification of key characteristics associated with different fault types [6]. A variety of studies have investigated feature extraction techniques grounded in time–frequency domain signal processing. These methods encompass approaches such as empirical mode decomposition (EMD) [7], ensemble empirical mode decomposition (EEMD) [8], local mean decomposition (LMD) [9], wavelet transform (WT), wavelet packet transform (WPT), empirical wavelet transform (EWT) [10], Variational Mode Decomposition (VMD) [11], multi-scale cross feature extraction module (MSCM) [12], and the frequency slice wavelet transform (FSWT) [13]. Among these methods, VMD has emerged as a highly promising approach due to its solid theoretical foundation and its ability to address limitations associated with EMD and its variants, such as mode mixing and lack of continuity. Unlike traditional methods, such as WT, WPT, and EWT, which require predefined basis functions, VMD is adaptive. It dynamically determines basis functions and partitions frequency bands based on spectral positions (center frequencies) and frequency scales (bandwidths). This adaptability allows VMD to effectively decompose signals with nonlinear and nonstationary characteristics, making it particularly suitable for analyzing vibration signals in rolling bearings [14]. By mitigating issues related to mode ambiguity and improving spectral resolution, VMD ensures robust and precise feature extraction, even for signals with overlapping frequency components or complex time–frequency structures [15].

Various research efforts have investigated the utilization of VMD for diagnosing faults in rolling bearings. For instance, ref. [14] investigated the modeling of rolling bearing fault simulation vibration signals and actual vibration signals, applying VMD to fault diagnosis. The extracted fault features at different locations were compared with those obtained using EMD, demonstrating the feasibility and superiority of VMD in rolling bearing fault detection. Several algorithms for parameter optimization have been proposed to tackle the challenge of manually setting the number of VMD modes and the associated penalty factor. These algorithms seek to improve VMD’s adaptability and accuracy by automatically determining optimal parameter values, which enhances decomposition performance and fault feature extraction [16,17,18]. Furthermore, ref. [19] proposed a fault information-guided VMD technique aimed at identifying weak, repetitive transient characteristics in bearings. This approach introduced the fault characteristic amplitude ratio and leveraged it to identify optimal bandwidth control parameters, thus enhancing the extraction of fault-related information. Similarly, ref. [20] introduced an adaptive signal decomposition method called Recursive Variational Mode Extraction. This technique dynamically selects the initial center frequency and penalty factor based on the dominant frequency of the residual signal from previous decomposition iterations, enabling the reconstruction of specific sub-components. The RVME method proved highly effective for diagnosing bearing faults due to its iterative refinement mechanism. In addition, various information metrics are utilized to evaluate and select the modes derived from the VMD decomposition of the data. These metrics include envelope spectral entropy [21], arrangement entropy [22], and the Pearson correlation coefficient [23], among others.

Despite its advantages, VMD exhibits certain limitations in practical applications. In rolling bearing fault feature extraction, localized damage in the bearing induces periodic impulsive characteristics within the vibration signal. These impulsive features are modulated by the bearing’s intrinsic frequencies, resulting in a series of resonance peaks. When utilizing Variational Mode Decomposition (VMD) to extract these resonance peaks, setting a high penalty factor can effectively suppress mode aliasing. However, this approach may simultaneously attenuate critical fault-related features. Conversely, employing a low penalty factor reduces suppression effects but increases the risk of aliasing, leading to modes contaminated by irrelevant interference components.

Despite numerous attempts to enhance fault feature extraction through modifications of VMD-based methods, these approaches have not fundamentally addressed the limitations imposed by the Wiener-filtered waveform. As a result, the filtering waveforms in existing improved VMD techniques remain inherently fixed, lacking the flexibility and adaptability required to effectively process signals with more diverse and complex bandwidth distributions. This rigidity poses a significant challenge for accurately capturing fault-related features, particularly in scenarios involving highly dynamic signal characteristics.

To address these limitations, ref. [24] proposed Variational Nonlinear Chirp Mode Decomposition (VNCMD), which leverages demodulation techniques and minimizes the bandwidth of demodulated baseband signals to analyze nonlinear chirp signals. While effective for broadband signals, VNCMD assumes that the modulating waveforms in chirp signals are inherently narrowband. This assumption limits its ability to efficiently decompose signals with modulating waveforms whose center frequencies deviate significantly from zero. Ref. [25] introduced the Generalized Variational Mode Decomposition method, which creates a series of variational models. Each model defines a unique constrained optimization problem for its corresponding mode. These problems are solved using an advanced multiplicative alternating direction technique, enabling multi-scale, fixed-frequency signal decomposition. This methodology enables the original signal to be decomposed into either multiple narrowband modes or a combination of several narrowband modes with one broadband mode based on specific requirements. Nevertheless, when faced with signals containing multiple broadband modes, the method encounters challenges, often resulting in decomposition failures in such cases.

To address these challenges, this study introduces a new algorithm, Variable-Filtered Waveform Variational Mode Decomposition (VFW-VMD). Recognizing that VMD fundamentally operates as a series of adaptive Wiener filters in the frequency domain, the VFW-VMD approach dynamically modifies the filtering waveform to adapt to signals with varying bandwidths. This dynamic adjustment effectively reduces mode aliasing and interference. The enhancement is achieved by altering the time-dependent bias of the target signal within the Tikhonov regularization term in the frequency–domain Wiener filter’s objective function. This novel technique significantly improves fault feature extraction for rolling bearings.

In practical industrial applications, the lightweight design and generalization capability of models are crucial to ensure their adaptability across diverse scenarios. For example, ref. [26] presents an energy-efficient mechanical fault diagnosis method using the neural-dynamics-inspired SpikingFormer (MSF) metric, which enables accurate fault recognition even with limited samples. Similarly, ref. [27] introduces a lightweight and precise approach based on a progressive joint-transfer ensemble network (PJTEN) and a Markov-lightweight strategy (MLS), and a Multi-source Domain-Class Gradient Coordination Meta-Learning (MDGCML) framework has been proposed in [28], which learns the generalized boundaries for all tasks by coordinating gradients across domains and classes. Building upon MDGCML, a joint learning paradigm involving parameter sharing between open-set and closed-set classifiers is established to enable rapid model adaptation to unknown domains. The proposed VFW-VMD model in this study serves as a universal approach, with its dynamic adjustment of mode bandwidth estimation further enhancing its generalization capability. This model is not only suitable for rolling bearing fault feature extraction but also applicable to cardiac signal analysis and seismic signal analysis. Moreover, VFW-VMD retains the ability of VMD to simultaneously extract multiple signal modes, avoiding the issue of sequential mode extraction encountered in improved VMD methods such as RVME, thereby ensuring the lightweight nature of the model.

This study aims to establish the mathematical foundation for VFW-VMD, explain the mechanism behind waveform filtering adjustments, and demonstrate its effectiveness. Specifically, the filtering waveform is tailored based on signal bandwidth to achieve the desired analytical outcomes. The paper is structured as follows: Section 2 presents a detailed review of the VMD method. Section 3 explores the mathematical principles of VFW-VMD and examines its convergence properties. Section 4 evaluates the performance of VFW-VMD through the analysis of both simulated and real-world signals. Finally, Section 5 offers a summary of the conclusions and suggests potential directions for future research.

## 2. Review of VMD

In VMD, the intrinsic mode functions (IMFs) are defined as amplitude modulation-frequency modulation (AM-FM) signals, as described in [11]:

(1) $$u_{k}\left(t\right)=a_{k}\left(t\right)cos\left(\phi_{k}\left(t\right)\right)$$

where, $a_{k}\left(t\right)$ represents the instantaneous amplitude(IA), $\phi_{k}\left(t\right)$ is the corresponding phase function, and its derivative, $\omega_{k}\left(t\right)=\phi_{k}^{\prime }\left(t\right)$, is the instantaneous frequency(IF). The condition $\phi_{k}^{\prime }\left(t\right)\geq 0$ must hold. Let $a_{k}\left(t\right)$ and $\omega_{k}\left(t\right)$ be non-negative smooth functions that satisfy the conditions $|a_{k}^{\prime }\left(t\right)|\ll |\omega \left(t\right)|$ and $|\omega_{k}^{\prime }\left(t\right)|\ll |\omega_{k}\left(t\right)|$. This implies that IAs and IFs change much more slowly than their respective phase functions [11]. Therefore, the IMFs are assumed to be band-limited signals [29].

Next, VMD introduced a frequency–domain representation of the Wiener filter for signals contaminated by additive zero-mean Gaussian noise:

(2) $$f_{0}=f+\eta$$

Reconstructing an unknown signal is a classic example of an ill-posed inverse problem. This challenge is typically addressed using the Tikhonov regularization method. In this approach, the rate of change in the signal waveform is often treated as a physical quantity closely related to the signal itself, so the regular term ${∥\partial_{t}f∥}_{2}^{2}$ is added, and the objective function is as follows:

(3) $$\min_{f}\left\{{∥f-f_{0}∥}_{2}^{2}+\alpha {∥\partial_{t}f∥}_{2}^{2}\right\}$$

The Euler–Lagrange equations can be easily derived and are typically solved in the Fourier domain [11]:

(4) $$\hat{f}\left(\omega \right)=\frac{{\hat{f}}_{0}}{1+\alpha \omega^{2}}$$

The Fourier transform of the signal f(t) is denoted as $\hat{f}\left(\omega \right)$. The reconstructed signal *f* is a low-pass, narrowband version of the original input signal $f_{0}$, centered at ω=0. The solution involves convolving with a Wiener filter, where α represents the variance of white noise. The signal is modeled with a low-pass power spectrum and a prior of $1/\omega^{2}$.

VMD estimates the bandwidth of each mode through a systematic process: (1) applying the Hilbert transform to obtain the analytic signal for each mode $u_{k}$, (2) shifting the spectrum to the baseband by modulating with an exponential function, and (3) calculating the bandwidth by minimizing the squared $L^{2}$ norm of the gradient of the demodulated signal.

To obtain optimal IMFs and fully decompose the input signal, the following minimization strategy is used:

(5) $$\begin{array}{cc} \min_{\left\{u_{k}\right\},\left\{\omega_{k}\right\}} & \left\{\alpha \sum_{k}{∥\partial_{t}\left[\left(\delta \left(t\right)+\frac{j}{\pi t}\right)*u_{k}\left(t\right)\right]e^{-j\omega_{k}t}∥}_{2}^{2}\right\}\\ s.t. & \;\sum_{k=1}^{K}u_{k}\left(t\right)=x\left(t\right) \end{array}$$

## 3. Proposed Method

### 3.1. Variable Filtered-Waveform Variational Mode Decomposition

Rolling bearing failures typically induce resonance within the system, resulting in fault characteristic signals that exhibit a specific excitation resonance frequency as the center frequency, accompanied by a series of sidebands or resonance peaks. VMD can be regarded as an ensemble of automatically optimized Wiener filters [11]. However, the bandwidth estimation method for modes limits the filter waveform’s adaptability to the frequency–domain characteristics of bearing fault signals. As a result, even with the correct number of modes, changes to the penalty factor can cause some loss of fault information or lead to the separation and aliasing of resonance peaks. To overcome this limitation, this paper introduces a Variable Filtered-Waveform Variational Mode Decomposition (VFW-VMD) method. As a generalized and flexible approach, VFW-VMD effectively resolves the issue in VMD where the filter waveform cannot be dynamically adjusted. This enhanced adaptability makes VFW-VMD particularly well suited for extracting fault features in rolling bearings while also demonstrating robust performance in the analysis of other broadband signals.

To enable the efficient decomposition of signals with varying bandwidths, it is essential to modify the bandwidth estimation approach employed in VMD. In this work, we propose replacing the squared $L_{2}$-norm of the gradient with the squared norm of an arbitrary fractional-order derivative. Specifically, the second term in (3) is reformulated as $\alpha {∥\frac{\partial^{l}f}{\partial t^{l}}∥}_{2}^{2}$, providing greater flexibility in adapting to signals with diverse spectral characteristics, thus (3) is rewritten in the following form:

(6) $$\min_{f}\left\{{∥f-f_{0}∥}_{2}^{2}+\alpha {∥\frac{\partial^{l}f}{\partial t^{l}}∥}_{2}^{2}\right\}$$

Ref. [30] presents the relationship between fractional order calculus and the Fourier transform, assuming *f* is a causal function. The following equation describes the connection between its fractional-order calculus and its Fourier transform:

(7) $$F\left[\frac{d^{q}f}{dt^{q}}\right]\equiv \int_{0}^{\infty }e^{-j\omega t}\frac{d^{q}f}{dt^{q}}dt$$

where *q* is any real number, (7) can be rewritten as

(8) $$\frac{d^{q}f}{dt^{q}}=F^{-1}\left\{{\left(j\omega \right)}^{q}F\left[f\right]\right\}$$

Thus, (6) is solved in the frequency domain:

(9) $$\hat{f}\left(\omega \right)=\frac{{\hat{f}}_{0}}{1+{\alpha |\omega |}^{2l}}$$

**Figure 1:**
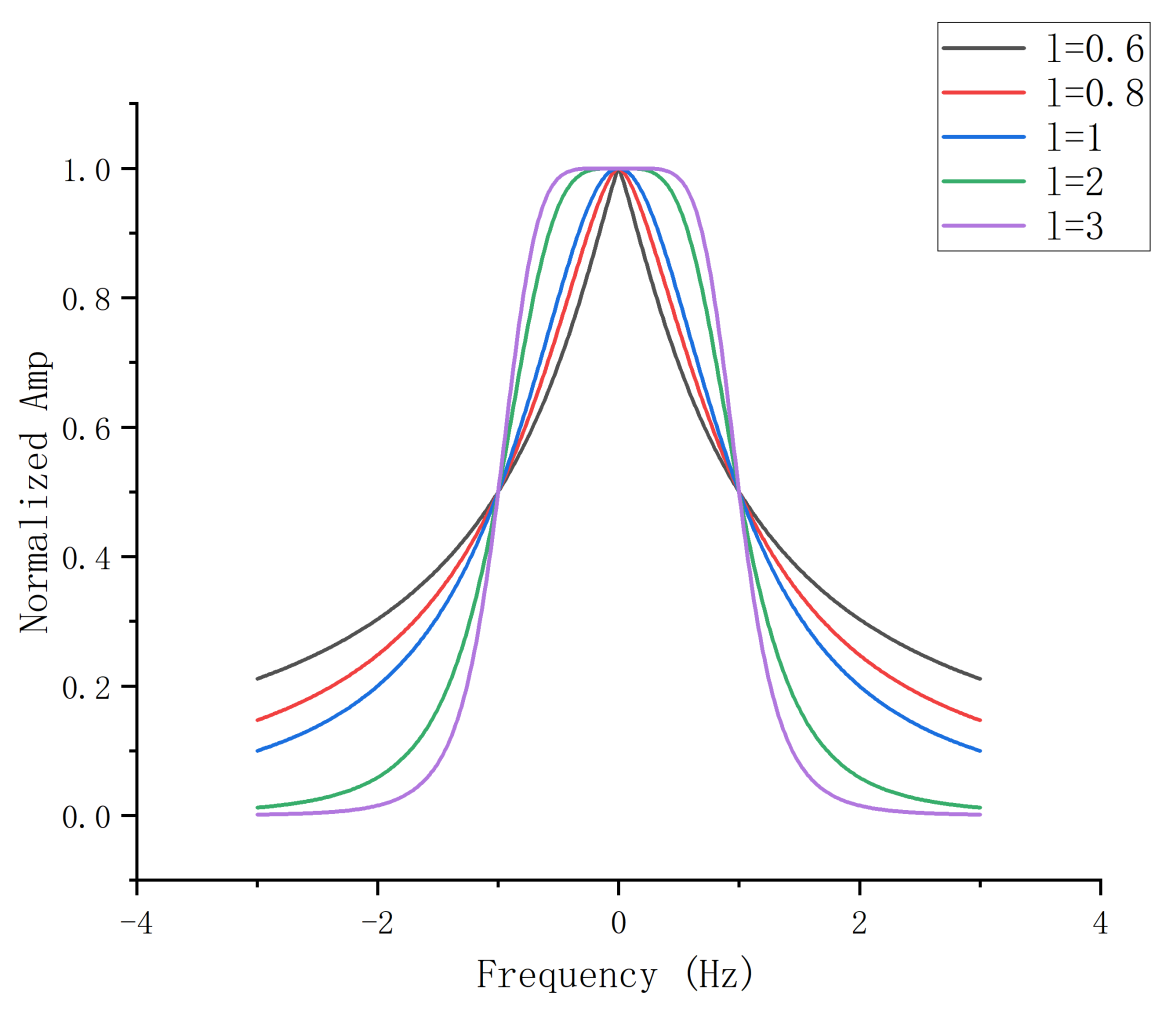
Wiener filter waveforms at different bias orders.

It is observed that different orders of bias in the time domain result in varying selections of low-pass narrow bands around the input signal $f_{0}$ near ω=0. Specifically, as illustrated in Figure 1, an increase in the decomposition order leads to a weaker filtering effect near the center frequency and a stronger effect further away from it, exhibiting enhanced edge-preserving capability. Consequently, the signal’s power spectral prior transitions from the low-pass form of $1/\omega^{2}$ to $1/\omega^{2l}$.

Building upon the decomposition framework of VMD, the following minimization problem is formulated based on the aforementioned improved Wiener filter:

(10) $$\begin{array}{cc} \min_{\left\{u_{k}\left(t\right)\right\},\left\{\omega_{k}\right\}} & \left\{\alpha \sum_{k=1}^{K}{∥\frac{\partial^{l}\left[\left(\delta \left(t\right)+\frac{j}{\pi t}\right)*u_{k}\left(t\right)\right]}{\partial t^{l}}e^{-j\omega_{k}t}∥}_{2}^{2}\right\}\\ s.t. & \sum_{k=1}^{K}u_{k}\left(t\right)=x\left(t\right) \end{array}$$

Solve the above optimization problem using augmented Lagrange multipliers as in VMD:

(11) $$L(\left\{u_{k}\left(t\right)\right\},\left\{\omega_{k}\right\},\lambda )=\alpha \sum_{k=1}^{K}{∥\frac{\partial^{l}\left[\left(\delta \left(t\right)+\frac{j}{\pi t}\right)*u_{k}\left(t\right)\right]}{\partial t^{l}}e^{-j\omega_{k}t}∥}_{2}^{2}+{∥x\left(t\right)-\sum_{k=1}^{K}u_{k}\left(t\right)∥}_{2}^{2}+\langle \lambda \left(t\right),x\left(t\right)-\sum_{k=1}^{K}u_{k}\left(t\right)\rangle$$

The original minimization problem (9) is solved by finding the saddle point of the augmented Lagrangian through iterative sub-optimization steps. This approach is known as the alternating direction method of multipliers (ADMM).

To update the modes, we begin by rewriting subproblem (11) as an equivalent minimization problem:

(12) $$u_{k}^{n+1}=\arg\min_{u_{k}\in X}\left\{\alpha {∥\frac{\partial^{l}\left[\left(\delta \left(t\right)+\frac{j}{\pi t}\right)*u_{k}\left(t\right)\right]}{\partial t^{l}}e^{-j\omega_{k}t}∥}_{2}^{2}+{∥f\left(t\right)-\sum_{i}u_{i}\left(t\right)+\frac{\lambda (t)}{2}∥}_{2}^{2}\right\}$$

For simplicity, we omit the superscripts ${•}^{n}$ and ${•}^{n+1}$ for the fixed directions $\omega_{k}$ and $u_{i\neq k}$, respectively, but each is understood to represent the most recent update. Using the Parseval/Plancherel Fourier isometry under the norm, this problem can now be solved in the spectral domain:

(13) $${\hat{u}}_{k}^{n+1}=\arg\min_{{\hat{u}}_{k}\in X}\left\{\alpha {∥{\left(j\omega \right)}^{l}\left[\left(1+\mathrm{sgn}\left(\omega +\omega_{k}\right)\right){\hat{u}}_{k}\left(\omega +\omega_{k}\right)\right]∥}_{2}^{2}+{∥\hat{f}\left(\omega \right)-\sum_{i}{\hat{u}}_{i}\left(\omega \right)+\frac{\hat{\lambda }\left(\omega \right)}{2}∥}_{2}^{2}\right\}$$

We now perform a change in variables in the first term:

(14) $${\hat{u}}_{k}^{n+1}=\arg\min_{{\hat{u}}_{k},u_{k}\in X}\left\{\alpha {∥{\left(j\left(\omega -\omega_{k}\right)\right)}^{l}\left[\left(1+\mathrm{sgn}\left(\omega \right)\right){\hat{u}}_{k}\left(\omega \right)\right]∥}_{2}^{2}+{∥\hat{f}\left(\omega \right)-\sum_{i}{\hat{u}}_{i}\left(\omega \right)+\frac{\hat{\lambda }\left(\omega \right)}{2}∥}_{2}^{2}\right\}$$

By utilizing the Hermitian symmetry property of real signals in the reconstruction fidelity term, both components can be represented as integrals over the non-negative half of the frequency spectrum:

(15) $$\begin{array}{cc} {\hat{u}}_{k}^{n+1} & =\arg\min_{{\hat{u}}_{k},u_{k}\in X}\left\{\int_{0}^{\infty }4\alpha {\left|\omega -\omega_{k}\right|}^{2l}{\left|{\hat{u}}_{k}\left(\omega \right)\right|}^{2}+2{\left|\hat{f}\left(\omega \right)-\sum_{i}{\hat{u}}_{i}\left(\omega \right)+\frac{\hat{\lambda }\left(\omega \right)}{2}\right|}^{2}d\omega \right\} \end{array}$$

The solution to this quadratic optimization problem is easily obtained by setting the first variation to zero for the positive frequencies:

(16) $${\hat{u}}_{k}^{n+1}\left(\omega \right)=\frac{\hat{f}\left(\omega \right)-\sum_{i\neq k}{\hat{u}}_{i}\left(\omega \right)+\frac{\hat{\lambda }\left(\omega \right)}{2}}{1+2\alpha {\left|\omega -\omega_{k}\right|}^{2l}}$$

This is clearly recognized as a variable filtered-waveform Wiener filtering of the current residual, with a signal prior of $1/|\omega -\omega_{k}{|}^{2l}$. The complete spectrum of the actual mode can be obtained through Hermitian symmetric extension. Meanwhile, the time–domain representation of the mode is reconstructed as the real component of the inverse Fourier transform applied to this filtered analytic signal.

The center frequencies $\omega_{k}$ are not present in the reconstruction fidelity term but appear only in the bandwidth prior. The corresponding problem is thus formulated as:

(17) $$\omega_{k}^{n+1}=\underset{\omega_{k}\in X}{\arg\;\min}\left\{\alpha {∥\frac{\partial^{l}\left[\left(\delta \left(t\right)+\frac{j}{\pi t}\right)*u_{k}\left(t\right)\right]}{\partial t^{l}}e^{-j\omega_{k}t}∥}_{2}^{2}\right\}$$

As before, the optimization can take place in the Fourier domain, and we end up optimizing

(18) $$\omega_{k}^{n+1}=\arg\min_{\omega_{k}}\left\{\int_{0}^{\infty }{\left|\omega -\omega_{k}\right|}^{2l}{\left|{\hat{u}}_{k}\left(\omega \right)\right|}^{2}d\omega \right\}$$

This quadratic problem is easily solved as

(19) $$\omega_{k}^{n+1}=\frac{\int_{0}^{\infty }\omega {\left|{\hat{u}}_{k}\left(\omega \right)\right|}^{\frac{2}{2l-1}}d\omega }{\int_{0}^{\infty }{\left|{\hat{u}}_{k}\left(\omega \right)\right|}^{\frac{2}{2l-1}}d\omega }$$

which makes the center frequency convergence performance of the new mode different from the decomposition order *l*. Clearly, (19) will fail when l≤0.5.

Therefore, the final VFW-VMD Algorithm 1 can be summarized as follows:
**Algorithm 1** VFW-VMD.**Initialize:** $\left\{{\hat{u}}_{k}^{1}\right\}$, $\left\{{\hat{\omega }}_{k}^{1}\right\}$, ${\hat{\lambda }}^{1}$, n←0 **repeat:**   1:n←n+1  2:**for** k=1:K **do**  3:   Update $u_{k}$ for all ω≥0:  4:   (20)$$\begin{array}{cc} & \;{\hat{u}}_{k}^{n+1}\left(\omega \right)=\frac{\hat{f}\left(\omega \right)-\sum_{i<k}{\hat{u}}_{i}^{n+1}\left(\omega \right)-\sum_{i>k}{\hat{u}}_{i}^{n}\left(\omega \right)+\frac{{\hat{\lambda }}^{n}\left(\omega \right)}{2}}{1+2\alpha {\left|\omega -\omega_{k}^{n}\right|}^{2l}} \end{array}$$  5:   Update $\omega_{k}$:  6:   (21)$$\begin{array}{cc} & \;\omega_{k}^{n+1}=\frac{\int_{0}^{\infty }\omega {\left|{\hat{u}}_{k}^{n+1}\left(\omega \right)\right|}^{\frac{2}{2l-1}}d\omega }{\int_{0}^{\infty }{\left|{\hat{u}}_{k}^{n+1}\left(\omega \right)\right|}^{\frac{2}{2l-1}}d\omega } \end{array}$$  7:**end for**  8:Dual ascent for all ω≥0:  9:(22)$$\begin{array}{cc} & \;{\hat{\lambda }}^{n+1}={\hat{\lambda }}^{n}+\tau \left[\hat{f}\left(\omega \right)-\sum_{k}{\hat{u}}_{k}^{n+1}\left(\omega \right)\right] \end{array}$$ **until convergence**: $\sum_{k}\frac{{∥{\hat{u}}_{k}^{n+1}-{\hat{u}}_{k}^{n}∥}_{2}^{2}}{{∥{\hat{u}}_{k}^{n}∥}_{2}^{2}}<\varepsilon$

It should be noted that both the VFW-VMD and VMD methods enforce constraints through Lagrange multipliers to achieve precise reconstruction of the original signal. For signals with intense noise, setting τ=0 releases the mandatory constraints of the Lagrange multipliers, enabling the model to perform filtering operations as shown in (9) while conducting mode decomposition. Unlike VMD, the filtering effect of VFW-VMD varies with the decomposition order *l*, as illustrated in Figure 1.

### 3.2. Convergence Analysis

In this section, we refer to [11] for the convergence analysis of VFW-VMD and evaluate its decomposition success rate and center frequency convergence performance.

Success rate analysis was first performed using the same artificial signals as in [11]:

(23) $$f_{Sig1}\left(t\right)=\cos\left(4\pi t\right)+\frac{1}{4}\cos\left(48\pi t\right)+\frac{1}{16}\cos\left(576\pi t\right)+\eta$$

where η∼N(0,σ) denotes Gaussian additive noise and σ controls the noise level by taking σ=0.1.

**Figure 2:**
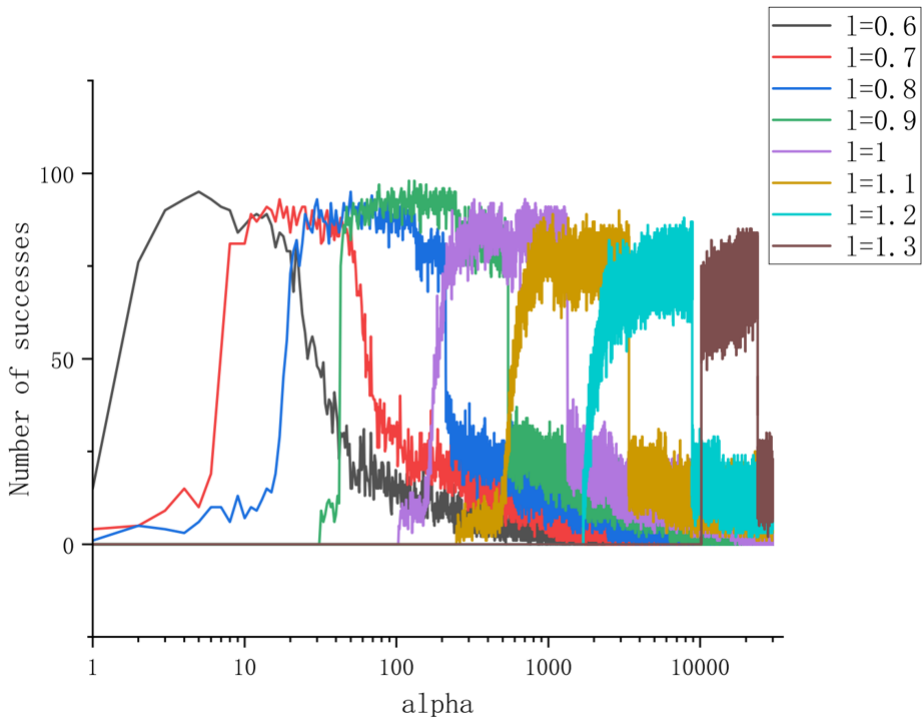
Success rate of VFW-VMD.

Figure 2 depicts the success rate of center frequency convergence under different penalty factors for VFW-VMD decomposition orders ranging from 0.6 to 1.3, with a step size of 0.1. For each penalty factor, the initial center frequencies are randomly distributed, and 100 experiments are conducted to assess the number of successful convergences. It is observed that as the decomposition order increases, the optimal penalty factor also increases, while the maximum success rate exhibits a slight decrease. Nevertheless, higher decomposition orders result in a broader range of penalty factors that yield a high success rate. Notably, the maximum success rate occurs at l=0.9 rather than at l=1, which corresponds to the VMD. This phenomenon is primarily influenced by (19), where larger spectral components carry more weight when *l* is small, and their contributions are progressively attenuated as *l* increases. Consequently, smaller values of *l* are more suitable for narrowband signals, whereas larger values of *l* are better suited for broadband signals.

Next, following the methodology outlined in [31], we verify whether VFW-VMD exhibits center frequency convergence properties comparable to those of VMD. The number of modes is set to 1, simplifying the model in (11) to

(24) $$\begin{array}{cc} L(u_{1},\omega_{1}) & =\alpha {∥\frac{\partial^{l}\left[\left(\delta \left(t\right)+\frac{j}{\pi t}\right)*u_{1}\left(t\right)\right]}{\partial t^{l}}e^{-j\omega_{1}t}∥}_{2}^{2}+{∥x\left(t\right)-u_{1}\left(t\right)∥}_{2}^{2} \end{array}$$

By minimizing $L(u_{1},\omega_{1})$, (16) and (19) are rewritten as

(25) $$u_{1}^{n+1}\left(\omega \right)=\frac{x(\omega )}{1+2\alpha {\left(\omega -\omega_{1}^{n}\right)}^{2l}}$$

(26) $$\omega_{1}^{n+1}=\frac{\int_{0}^{\infty }\omega {\left(u_{1}^{n+1}\left(\omega \right)\right)}^{\frac{2}{2l-1}}d\omega }{\int_{0}^{\infty }{\left(u_{1}^{n+1}\left(\omega \right)\right)}^{\frac{2}{2l-1}}d\omega }$$

Substituting (25) in (26), we obtain

(27) $$\omega_{1}^{n+1}=\frac{\int_{0}^{\infty }\omega {\left(\frac{x(\omega )}{1+2\alpha {\left(\omega -\omega_{1}^{n}\right)}^{2l}}\right)}^{\frac{2}{2l-1}}d\omega }{\int_{0}^{\infty }{\left(\frac{x(\omega )}{1+2\alpha {\left(\omega -\omega_{1}^{n}\right)}^{2l}}\right)}^{\frac{2}{2l-1}}d\omega }$$

Similarly verify that when $\omega_{1}^{n}<\omega_{1}$,

(28) $$\omega_{1}^{n}<\frac{\int_{0}^{\infty }\omega {\left(x\left(\omega \right)FB\left(\omega ,\omega_{1}^{n}\right)\right)}^{\frac{2}{2l-1}}d\omega }{\int_{0}^{\infty }{\left(x\left(\omega \right)FB\left(\omega ,\omega_{1}^{n}\right)\right)}^{\frac{2}{2l-1}}d\omega }<\omega_{1}$$

where $\omega_{1}$ represents the optimal center frequency (CF) of the target mode, which is located in a local region of the Fourier spectrum x(ω). Additionally, $FB\left(\omega ,\omega_{1}^{n}\right)$ is defined by the following equation:

(29) $$FB\left(\omega ,\omega_{1}^{n}\right)=\frac{1}{1+2\alpha {\left(\omega -\omega_{1}^{n}\right)}^{2l}}$$

It is easy to know that $FB\left(\omega ,\omega_{1}^{n}\right)$ conforms to

(30) $$\left\{\begin{array}{cc} FB\left(\omega ,\omega_{1}^{n}\right) & =FB\left(2\omega_{1}^{n}-\omega ,\omega_{1}^{n}\right)\\ FB\left(\omega_{a},\omega_{1}^{n}\right) & <FB\left(\omega_{b},\omega_{1}^{n}\right),\;\omega_{a}<\omega_{b}<\omega_{1}^{n}\\ FB\left(\omega_{a},\omega_{1}^{n}\right) & >FB\left(\omega_{b},\omega_{1}^{n}\right),\;\omega_{1}^{n}<\omega_{a}<\omega_{b} \end{array}\right.$$

For convenience, the integral region of the numerator in the intermediate term of (28) is adjusted due to the limited bandwidth of the target mode:

(31) $$\begin{array}{cc} \; & \int_{0}^{\infty }\omega {\left(x\left(\omega \right)FB\left(\omega ,\omega_{1}^{n}\right)\right)}^{\frac{2}{2l-1}}d\omega =\int_{\omega_{1}^{n}-\Delta \omega }^{\omega_{1}^{n}+\Delta \omega }\omega {\left(x\left(\omega \right)FB\left(\omega ,\omega_{1}^{n}\right)\right)}^{\frac{2}{2l-1}}d\omega \end{array}$$

Since Δω significantly exceeds the semi-bandwidth of the target modes, (31) can be naturally expressed as

(32) $$\begin{array}{cc} \; & \int_{\omega_{1}^{n}-\Delta \omega }^{\omega_{1}^{n}+\Delta \omega }\omega {\left(x\left(\omega \right)FB\left(\omega ,\omega_{1}^{n}\right)\right)}^{\frac{2}{2l-1}}d\omega =\int_{\omega_{1}^{n}-\Delta \omega }^{\omega_{1}^{n}}\omega {\left(x\left(\omega \right)FB\left(\omega ,\omega_{1}^{n}\right)\right)}^{\frac{2}{2l-1}}d\omega \\ \; & +\int_{\omega_{1}^{n}}^{\omega_{1}^{n}+\Delta \omega }\omega \left(x^{\frac{2}{2l-1}}\left(2\omega_{1}^{n}-\omega \right)FB^{\frac{2}{2l-1}}\left(\omega ,\omega_{1}^{n}\right)\right)d\omega \\ \; & +\int_{\omega_{1}^{n}}^{\omega_{1}^{n}+\Delta \omega }\omega \left[x^{\frac{2}{2l-1}}\left(\omega \right)-x^{\frac{2}{2l-1}}\left(2\omega_{1}^{n}-\omega \right)\right]FB^{\frac{2}{2l-1}}\left(\omega ,\omega_{1}^{n}\right)d\omega \end{array}$$

The initial two terms in (32) can be further reduced to

(33) $$\begin{array}{cc} \; & \int_{\omega_{1}^{n}-\Delta \omega }^{\omega_{1}^{n}}\omega {\left(x\left(\omega \right)FB\left(\omega ,\omega_{1}^{n}\right)\right)}^{\frac{2}{2l-1}}d\omega +\int_{\omega_{1}^{n}}^{\omega_{1}^{n}+\Delta \omega }\omega \left(x^{\frac{2}{2l-1}}\left(2\omega_{1}^{n}-\omega \right)FB^{\frac{2}{2l-1}}\left(\omega ,\omega_{1}^{n}\right)\right)d\omega \\ \; & =2\omega_{1}^{n}\int_{\omega_{1}^{n}-\Delta \omega }^{\omega_{1}^{n}}{\left(x\left(\omega \right)FB\left(\omega ,\omega_{1}^{n}\right)\right)}^{\frac{2}{2l-1}}d\omega \end{array}$$

Then, (32) is rewritten as

(34) $$\begin{array}{cc} \; & \int_{\omega_{1}^{n}-\Delta \omega }^{\omega_{1}^{n}+\Delta \omega }\omega {\left(x\left(\omega \right)FB\left(\omega ,\omega_{1}^{n}\right)\right)}^{\frac{2}{2l-1}}d\omega =2\omega_{1}^{n}\int_{\omega_{1}^{n}-\Delta \omega }^{\omega_{1}^{n}}{\left(x\left(\omega \right)FB\left(\omega ,\omega_{1}^{n}\right)\right)}^{\frac{2}{2l-1}}d\omega +\\ \; & \int_{\omega_{1}^{n}}^{\omega_{1}^{n}+\Delta \omega }\omega \left[x^{\frac{2}{2l-1}}\left(\omega \right)-x^{\frac{2}{2l-1}}\left(2\omega_{1}^{n}-\omega \right)\right]FB^{\frac{2}{2l-1}}\left(\omega ,\omega_{1}^{n}\right)d\omega \end{array}$$

Likewise, the denominator in the intermediate term of (34) is reformulated as

(35) $$\begin{array}{cc} \; & \int_{\omega_{1}^{n}-\Delta \omega }^{\omega_{1}^{n}+\Delta \omega }{\left(x\left(\omega \right)FB\left(\omega ,\omega_{1}^{n}\right)\right)}^{\frac{2}{2l-1}}d\omega =2\int_{\omega_{1}^{n}-\Delta \omega }^{\omega_{1}^{n}}{\left(x\left(\omega \right)FB\left(\omega ,\omega_{1}^{n}\right)\right)}^{\frac{2}{2l-1}}d\omega +\\ \; & \int_{\omega_{1}^{n}}^{\omega_{1}^{n}+\Delta \omega }\left[x^{\frac{2}{2l-1}}\left(\omega \right)-x^{\frac{2}{2l-1}}\left(2\omega_{1}^{n}-\omega \right)\right]FB^{\frac{2}{2l-1}}\left(\omega ,\omega_{1}^{n}\right)d\omega \end{array}$$

Generally, the target mode within the Fourier spectrum x(ω) can be roughly treated as a symmetric spectrum. Thus, (36) is derived when $\omega_{1}^{n}<\omega_{1}$, with the integral region of the second term in (35) centered at $\omega_{1}$ and confined to a limited bandwidth:

(36) $$\left|x(\omega )\right|>\left|x\left(2\omega_{1}^{n}-\omega \right)\right|$$

Thus, the following result can be expressed as

(37) $$\begin{array}{cc} \; & \int_{\omega_{1}^{n}}^{\omega_{1}^{n}+\Delta \omega }\omega \left[x^{\frac{2}{2l-1}}\left(\omega \right)-x^{\frac{2}{2l-1}}\left(2\omega_{1}^{n}-\omega \right)\right]FB^{\frac{2}{2l-1}}\left(\omega ,\omega_{1}^{n}\right)d\omega \\ \; & >\omega_{1}^{n}\int_{\omega_{1}^{n}}^{\omega_{1}^{n}+\Delta \omega }\left[x^{\frac{2}{2l-1}}\left(\omega \right)-x^{\frac{2}{2l-1}}\left(2\omega_{1}^{n}-\omega \right)\right]FB^{\frac{2}{2l-1}}\left(\omega ,\omega_{1}^{n}\right)d\omega \end{array}$$

By combining Equations (37)–(39), the left-hand inequality of Equation (32) can be obtained as

(38) $$\omega_{1}^{n}<\frac{\int_{\omega_{1}^{n}-\Delta \omega }^{\omega_{1}^{n}+\Delta \omega }\omega {\left(x\left(\omega \right)FB\left(\omega ,\omega_{1}^{n}\right)\right)}^{\frac{2}{2l-1}}d\omega }{\int_{\omega_{1}^{n}-\Delta \omega }^{\omega_{1}^{n}+\Delta \omega }{\left(x\left(\omega \right)FB\left(\omega ,\omega_{1}^{n}\right)\right)}^{\frac{2}{2l-1}}d\omega }$$

By replacing $\omega_{1}^{n}$ with $\omega_{1}$ in the integral regions of Equations (34) and (38), the right-hand inequality of Equation (31) can also be demonstrated using the aforementioned method.

Similarly, we can prove that when $\omega_{1}^{n}>\omega_{1}$:

(39) $$\omega_{1}<\frac{\int_{0}^{\infty }\omega {\left(x\left(\omega \right)FB\left(\omega ,\omega_{1}^{n}\right)\right)}^{\frac{2}{2l-1}}d\omega }{\int_{0}^{\infty }{\left(x\left(\omega \right)FB\left(\omega ,\omega_{1}^{n}\right)\right)}^{\frac{2}{2l-1}}d\omega }<\omega_{1}^{n}$$

From the above proofs, it can be seen that VFW-VMD share the same tendency of center frequency convergence inherent in VMD. We set δ to be a tiny increment of the decomposition order *l*. The following is easily obtained:

(40) $$\begin{array}{cc} \; & x^{\frac{2}{2(l-\delta )-1}}\left(\omega \right)-x^{\frac{2}{2(l-\delta )-1}}\left(2\omega_{1}^{n}-\omega \right)>x^{\frac{2}{2l-1}}\left(\omega \right)-x^{\frac{2}{2l-1}}\left(2\omega_{1}^{n}-\omega \right)>x^{\frac{2}{2(l+\delta )-1}}\left(\omega \right)-x^{\frac{2}{2(l+\delta )-1}}\left(2\omega_{1}^{n}-\omega \right) \end{array}$$

From (40), it can be inferred that the center frequency convergence is faster for smaller values of *l* and slower for larger values of *l*. This conjecture will be further validated using a bearing failure simulation signal:

(41) $$f_{Sig2}\left(t\right)=x_{1}\left(t\right)+x_{2}\left(t\right)+x_{3}\left(t\right)+\eta$$

where σ=0.1, the expression for x(t) is

(42) $$x\left(t\right)=\sum_{j=1}^{N}Ae^{-\beta (t-jT)}\cos\left(2\pi \omega \left(t-jT+\theta \right)\right)u\left(t-jT\right)$$

where *A* denotes the amplitude, β denotes the damping coefficient, *T* denotes the failure period, ω denotes the structural resonance frequency, θ denotes the random slip of the bearing balls; here, we take a random number within 0 to 2π, and u(t) denotes the unit step function. The specific parameters are shown in Table 1.

**Figure 3:**
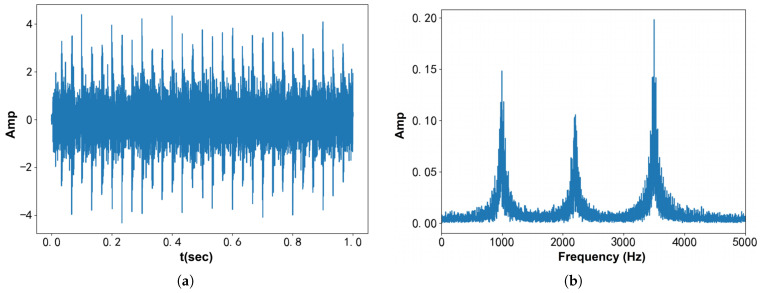
Bearing failure simulation signal: (**a**) Time–domain waveform. (**b**) Frequency spectrum.

**Figure 4:**
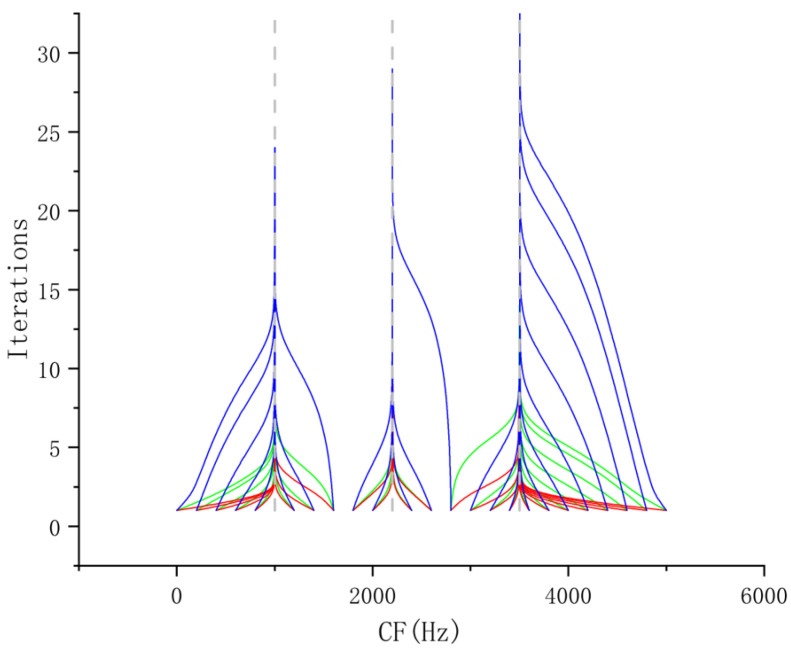
Convergence curve of center frequencies for VFW-VMD (Green: VMD, Red: VFW-VMD (l=0.8), Blue: VFW-VMD (l=1.2)).

Figure 3 presents the time–domainin and frequency-domain spectra of $f_{Sig2}\left(t\right)$. The shock component is clearly visible in the time–domain spectrum, while three distinct resonance peaks are observed in the frequency-domain spectrum. Figure 4 illustrates the center frequency update curves for different initial center frequencies with K=1. The green curve represents VMD, the red curve corresponds to l=0.8, and the blue curve corresponds to l=1.2, with the gray dotted line indicating the optimal center frequency. As shown in the figure, the curve distributions are consistent with the previous inference.

## 4. Simulation and Experimental Results

This section evaluates the performance of the proposed VFW-VMD algorithm by testing it on a range of signals and comparing its outcomes with those generated by VMD. The test signals include both simple and intricate structures, such as fixed-frequency components, signals with abrupt instantaneous frequency shifts from low to high, intrawave frequency modulation, and chirp signals. All computations are performed using Python 3.11.4.

### 4.1. Setting the Parameters in VMD and VFW-VMD

In VMD, proper configuration of the total number of modes (*K*) and the penalty factor (α) is crucial. Assuming *P* denotes the actual number of modes in the signal, optimal outcomes are achieved when K=P. Thus, all VMD simulations presume that *P* is known and set *K* accordingly. Additionally, α is chosen to ensure the optimal performance of VMD. While Lagrangian multipliers enable the accurate reconstruction of the input signal under low-noise conditions, they may hinder convergence when noise levels are high. As a result, the update parameter for λ, represented by τ, is set to zero, assuming the signal is noise-affected. For all simulations, except for *l* and α, the parameter settings for VFW-VMD remain consistent with those used in VMD.

### 4.2. Method Validation Based on Artificial Signals and Simulation Results

The outcomes of VMD and VFW-VMD are compared using four synthetic signals. The first signal features intrawave frequency modulation, defined as follows:

(43) $$\begin{array}{cc} \; & f_{Sig3}\left(t\right)=\frac{1}{1.2+\mathrm{Cos}(2\pi t)}+\left[\frac{1}{1.5+\mathrm{Sin}(2\pi t)}\times \mathrm{Cos}\left(32\pi t+0.2\mathrm{Cos}\left(64\pi t\right)\right)\right] \end{array}$$

**Figure 5:**
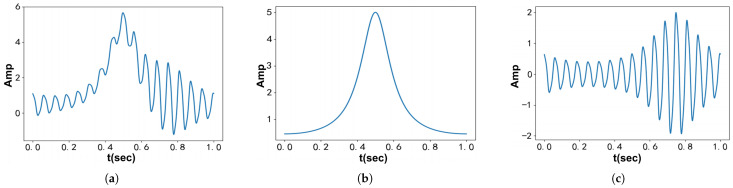
Time–domain representations of $f_{Sig3}\left(t\right)$ and its components: (**a**) Test signal $f_{Sig3}\left(t\right)$. (**b**) The first constituent component. (**c**) The second constituent component.

Figure 5 illustrates the signal and its modes. The first mode represents a tonal component, primarily exhibiting low-pass behavior. In contrast, the second mode shows significant intrawave frequency modulation, with a dominant peak at 16 Hz. This modulation generates several higher-order harmonics within the frequency spectrum. Notably, the second mode’s characteristics deviate significantly from the narrowband assumption of VMD, suggesting potential difficulties in isolating it accurately with this method.

**Figure 6:**
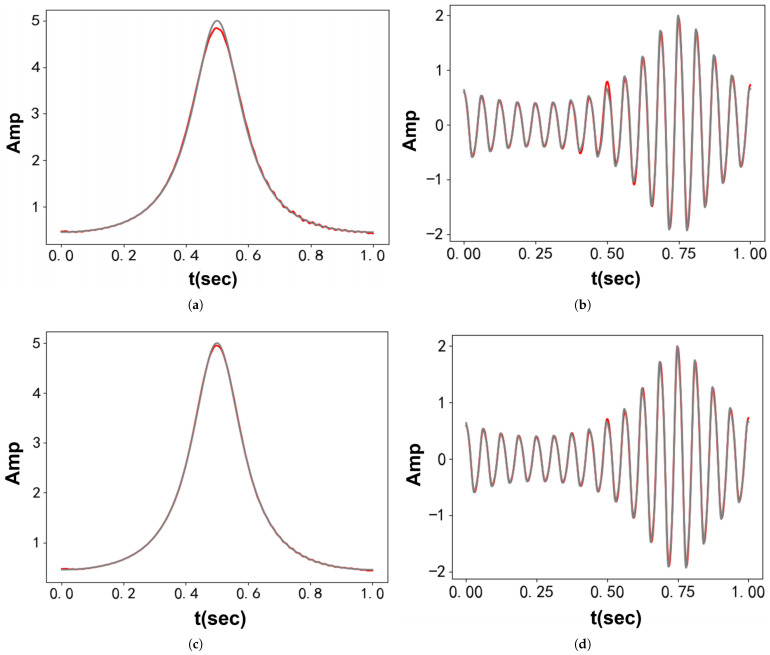
Decomposition results obtained using VMD and VFW-VMD (red: decomposed results; gray: original signal): (**a**) First mode obtained using VMD. (**b**) Second mode obtained using VMD. (**c**) First mode obtained using VFW-VMD. (**d**) Second mode obtained using VFW-VMD.

Figure 6 displays the results from VMD and VFW-VMD. In the VMD case, the higher-order harmonics are not solely assigned to the second mode. Instead, they are distributed between both modes, causing slight ripples in the first mode. For VFW-VMD, with l=3, the variation in the filtering waveform allows for the inclusion of more sideband harmonic components. Consequently, compared with VMD, VFW-VMD achieves improved decomposition performance. Table 2 summarizes the decomposition errors for both methods.

The second synthetic signal combines two frequency components: the first is a chirp signal, while the second undergoes a rapid frequency shift at the midpoint:

(44) $$\begin{array}{cc} f_{\mathrm{Sig}4}\left(t\right) & =2\cos\left(10\pi t+10\pi t^{2}\right)+\left\{\begin{array}{cc} \cos(60\pi t), & t\leq 0.5\\ \cos(100\pi t+10\pi ), & t>0.5 \end{array}\right. \end{array}$$

**Figure 7:**
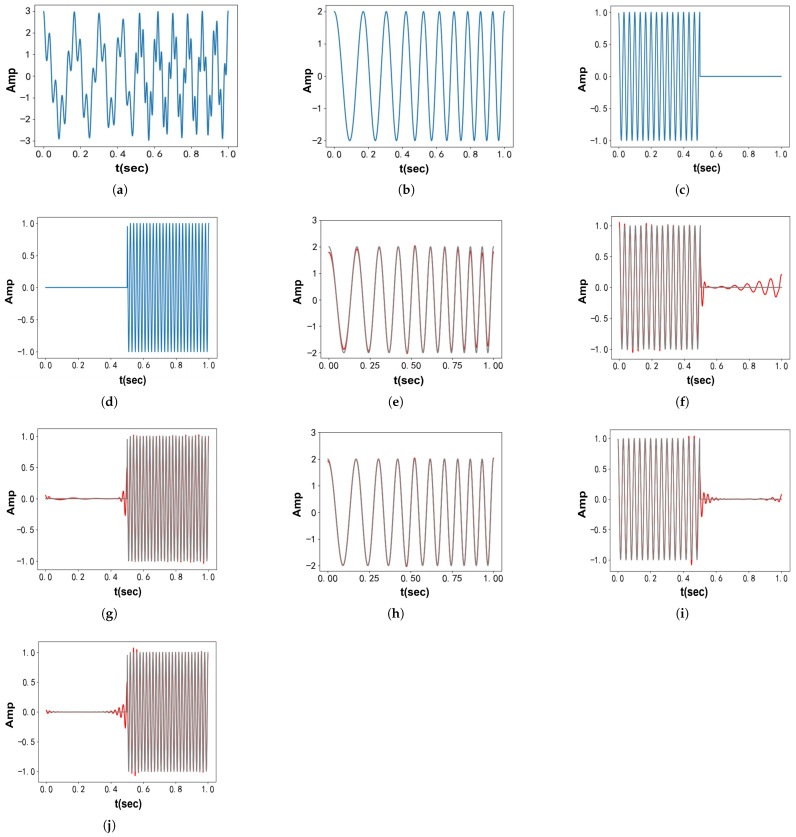
Time–domain representations of $f_{Sig4}\left(t\right)$ and its components, along with decomposition results obtained using VMD and VFW-VMD (red: decomposed results; gray: original signal): (**a**) Artificial signal $f_{Sig4}$. (**b**) The 1st component of $f_{Sig4}$. (**c**) The 2nd component of $f_{Sig4}$. (**d**) The 3rd component of $f_{Sig4}$. (**e**) Obtained the 1st mode using VMD. (**f**) Obtained the 2nd mode using VMD. (**g**) Obtained the 3rd mode using VMD. (**h**) Obtained the 1st mode using VFW-VMD. (**i**) Obtained the 2nd mode using VFW-VMD. (**j**) Obtained the 3rd mode using VFW-VMD.

Figure 7 displays the time–domain waveforms of $f_{Sig4}\left(t\right)$ and its components. Figure 7a–d show the waveforms, while Figure 7e–g present the decomposition results from VMD. Figure 7h–j show the decomposition results from VFW-VMD (l=2). Due to the wide bandwidth of chirp signals, which challenges the narrowband assumption of VMD, VFW-VMD clearly outperforms VMD in this case.

The third artificial signal is a superposition of two different chirp signals, which we chose because the first constituent component of this signal conflicts with our previous definition of mode, and it does not satisfy the condition $\phi_{k}^{\prime }\left(t\right)\geq 0$, as shown in the following equation:

(45) $$f_{\mathrm{Sig}5}\left(t\right)=\mathrm{Cos}\left(150\pi t-100\pi t^{2}\right)+\mathrm{Cos}\left(420\pi t-100\pi t^{2}\right)$$

**Figure 8:**
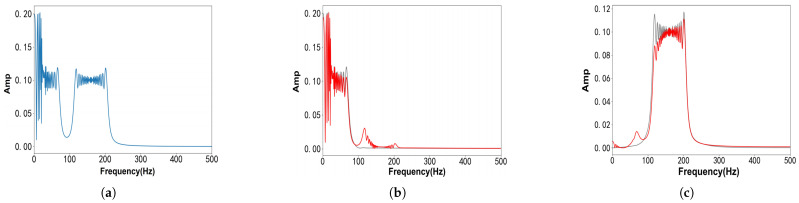
Frequency spectrum of sig5 and its decomposition results obtained using VMD and VFW-VMD (red: VMD; gray: VFW-VMD): (**a**) $f_{Sig5}\left(t\right)$ frequency spectrum. (**b**) 1st mode frequency spectrum using VMD and VFW-VMD. (**c**) 2nd mode frequency spectrum using VMD and VFW-VMD.

As shown in Figure 8, we show the results of the decomposition of $f_{Sig5}\left(t\right)$. Figure 8a depicts the frequency-domain representation of $f_{Sig5}\left(t\right)$, where both components exhibit wideband characteristics that differ significantly from the classical Wiener filter-based frequency-domain waveforms assumed in the VMD framework. Figure 8b,c present the decomposition results for the first and second components of $f_{Sig5}\left(t\right)$, respectively. The green curves correspond to the results obtained using the VFW-VMD method, while the red curves represent the results produced by the conventional VMD. It is evident that the VMD method suffers from severe mode aliasing and signal distortion. In contrast, the VFW-VMD method, which incorporates adjustments to the Wiener filter waveforms, achieves significantly improved decomposition results, effectively mitigating mode aliasing and preserving signal fidelity.

VNCMD is an extension of the VMD framework for the mode decomposition of nonlinear chirp signals. In VNCMD, a nonlinear chirp signal is defined as $g\left(t\right)=a\left(t\right)\cos\left(2\pi \int_{0}^{t}f\left(s\right)ds+\phi \right)$, where a(t) is defined to have a narrow band characteristic with frequency equal to the neighborhood of 0. If the main frequency of c becomes too far away from the origin of the separation frequency axis, VNCMD will not be able to decompose well. Next, we illustrate the advantages of VFW-VMD over VNCMD by using the fourth artificial signal:

(46) $$\begin{array}{cc} f_{\mathrm{Sig}6}\left(t\right) & =\mathrm{Cos}\left(70\pi t\right)\times \mathrm{Cos}(2\pi \left(0.2+100\pi t+50\pi t^{2}\right))\\ \; & +\mathrm{Cos}\left(70\pi t\right)\times \mathrm{Cos}(2\pi \left(0.2+300\pi t+50\pi t^{2}\right)) \end{array}$$

**Figure 9:**
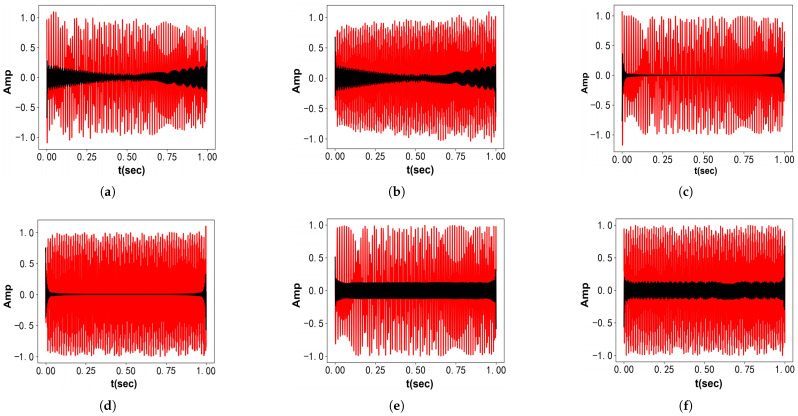
(**a**) First mode using VMD. (**b**) Second mode using VMD. (**c**) First mode using VFW-VMD. (**d**) Second mode using VFW-VMD. (**e**) First mode using VNCMD. (**f**) Second mode using VNCMD.

Figure 9 presents the decomposition results obtained using VMD, VFW-VMD (l=4), and VNCMD. In these subfigures, the red curves represent the estimated modes, while the black curves denote the estimation errors. It is evident that the decomposition performance of VFW-VMD is significantly superior to that of both VMD and VNCMD. Furthermore, due to the violation of the narrowband assumption of both VMD and VNCMD by $f_{Sig6}\left(t\right)$, complete decomposition of the signal cannot be achieved. However, VFW-VMD, by adjusting l=4, aligns the filtered waveform with the signal characteristics, resulting in minimal decomposition error.

### 4.3. Real-World Signals and Simulation Results

To evaluate the effectiveness of the proposed method for signal decomposition in practical applications, this study simulates an outer ring fault in the bearing of a circulating water pump. A scaled experimental setup was constructed to replicate this fault scenario by replacing the rolling bearing’s outer ring with a defective component. The experiment was specifically designed to investigate rolling bearing failures in circulating water pumps. The details of the experimental apparatus, including component selection and installation methods, are provided below.

The circulating water pump utilized in the experiment is a vertical centrifugal pump. The fault testing system primarily consists of hardware equipment and a data acquisition system. The hardware setup includes a circulating water circuit, motor, circulating water pump, regulating valve, water storage junction box, instrumentation and control platform, and associated pipelines. Vibration data associated with the outer ring fault were collected using vibration sensors and a signal acquisition demodulator.

**Figure 10:**
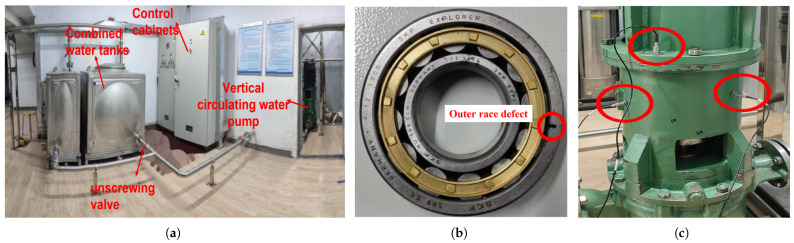
Experimental equipment: (**a**) Test stand. (**b**) Bearing with outer race failure. (**c**) Sensors layout.

Figure 10a provides an overview of the scaled test bench for the circulating water pump. The defective bearing used in the outer race fault experiment, featuring artificial defects created via electrical discharge machining, is displayed in Figure 10b. These defects measure 1.2 mm in depth and 0.9 mm in width. As illustrated in Figure 10c, acceleration sensors are positioned horizontally and vertically on the bearing block to capture vibration signals. The performance specifications of the centrifugal pump and the ball bearings employed in the experiment (DALIANSIFANG MOTOR-PUMP Co., Ltd., Dalian, China) are detailed in Table 3 and Table 4, respectively.

**Figure 11:**
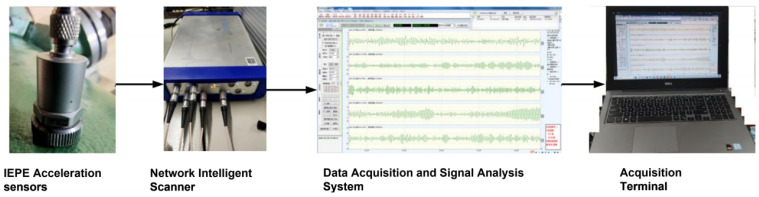
Vibration signal acquisition module.

The hardware equipment for acquiring the vibration signal of the pump body includes a magnetic suction acceleration sensor (mounted in the circumferential direction of the pump casing), a networked collector, a compact insulated magnetic base, shielded cable wires, and a data acquisition and signal analysis system. The specific parameters are provided in Table 5. Figure 11 illustrates the technical framework of the vibration signal acquisition module, with a sampling frequency of 20 kHz and an outer ring failure frequency of 135 Hz [32].

**Figure 12:**
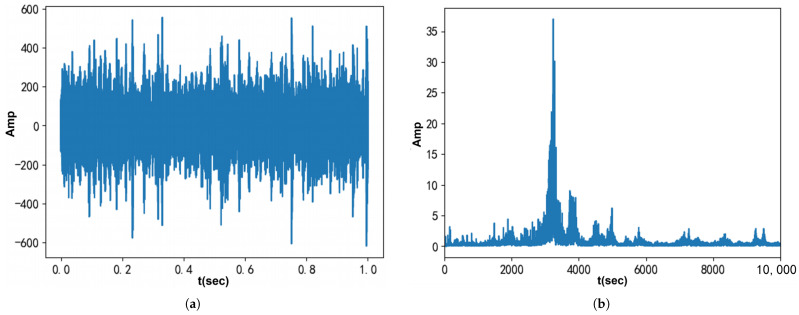
Measured vibration signal from the bearing with an outer race fault: (**a**) Time waveform. (**b**) Fourier spectrum.

**Figure 13:**
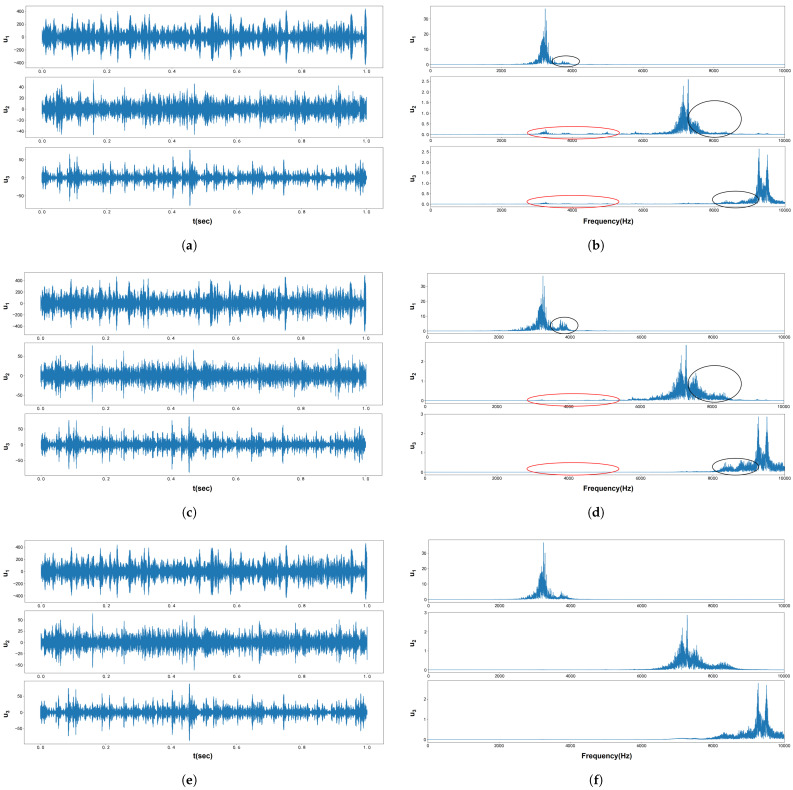
Figure 12 decomposition results: (**a**) VMD decomposition modes. (**b**) Spectrum of VMD decomposition modes. (**c**) VFW-VMD decomposition modes. (**d**) Spectrum of VFW-VMD decomposition modes. (**e**) VNCMD decomposition modes. (**f**) Spectrum of VNCMD decomposition modes.

**Figure 14:**
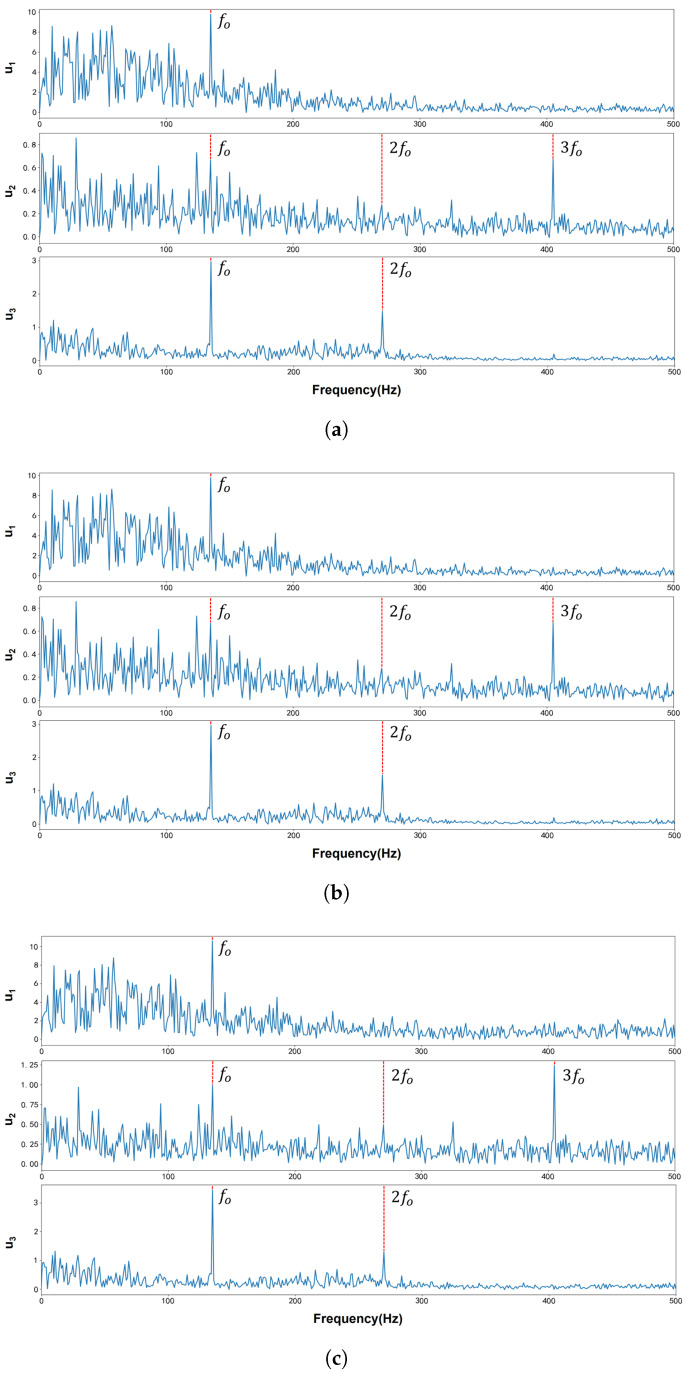
Mode envelope spectra derived from the decomposition of bearing outer ring fault signals using VMD and VFW-VMD (red: VMD; gray: VFW-VMD): (**a**) Envelope spectrums of the VMD decomposition modes. (**b**) Envelope spectrums of the VFW-VMD decomposition modes. (**c**) Envelope spectrums of the VNCMD decomposition modes.

Figure 12 displays the waveform and Fourier spectrum of the original bearing fault vibration signal, which was collected by the accelerometer. According to the method outlined in [31], the signal contains three modes, with center frequencies of 3215 Hz, 7132 Hz, and 9340 Hz, respectively. These center frequencies served as the initial values for VMD, VFW-VMD, and VNCMD. The penalty factors were fine-tuned to achieve the optimal decomposition of the extracted modes. The results in both the time and frequency domains are shown in Figure 13, while the envelope spectra are presented in Figure 14.

**Figure 15:**
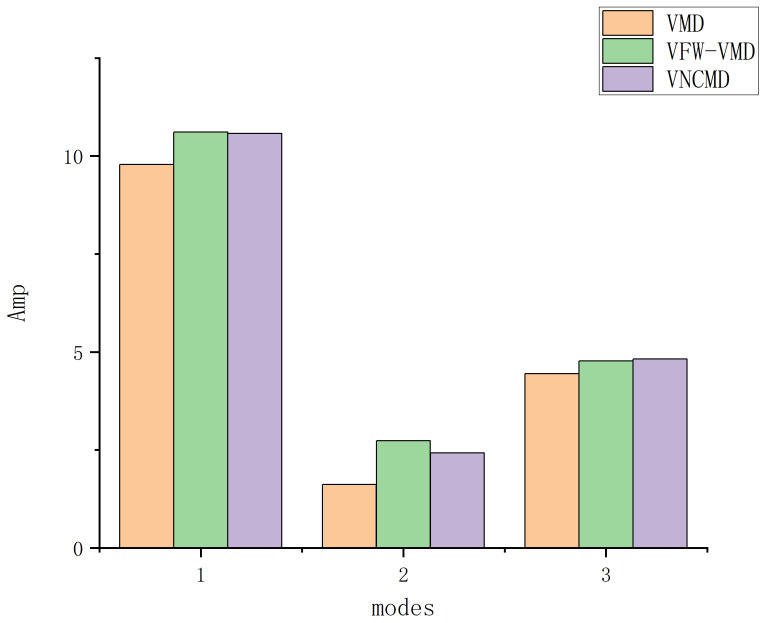
The sum of modes fault characteristic frequencies and harmonic amplitudes obtained by using VMD, VFW-VMD, and VNCMD.

**Figure 16:**
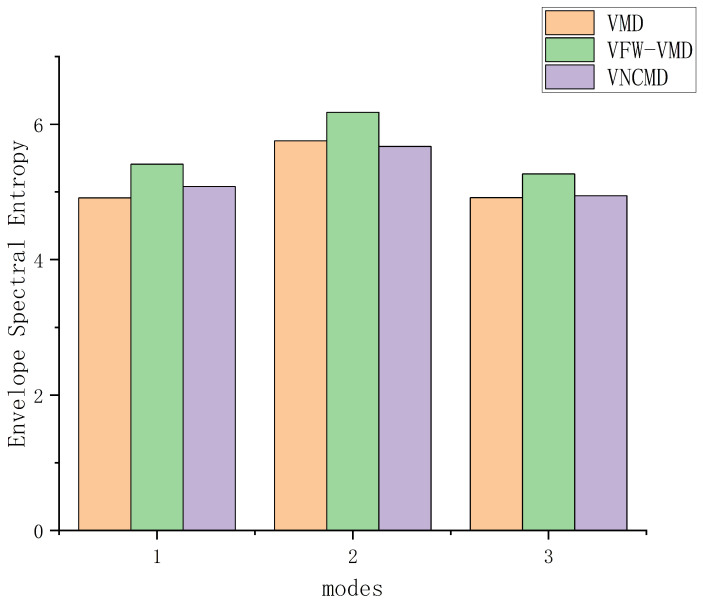
The envelope spectral entropy values of modes obtained by using VMD, VFW-VMD, and VNCMD.

As shown in Figure 13, compared with VMD, VFW-VMD exhibits a less pronounced mode aliasing effect (indicated by the red elliptical circle) and preserves mode components more completely (indicated by the black elliptical circle). Figure 15 presents the envelope spectrum fault characteristic frequency and its harmonic amplitude summation marked by the red dashed line in Figure 14. It can be observed that VMD extracts the least fault characteristic information, while VFW-VMD performs slightly better than VNCMD. Figure 16 illustrates the envelope spectral entropy of the modes extracted by the three methods. It can be observed that VFW-VMD retains the most fault feature information, but it also exhibits the highest envelope spectral entropy. This is attributed to the alteration of the filtered waveform, which, while preserving more complete spectral information near the central frequency of the modes, inevitably introduces additional noise. This issue can be mitigated using subsequent techniques such as wavelet denoising or stochastic resonance, which, however, fall beyond the scope of this study and are not further elaborated here.

## 5. Conclusions

This study proposes a VFW-VMD model as an improvement to the standard VMD. The aim is to address the limitation of fixed classical Wiener filtering waveforms in VMD, which hinders its adaptability to signals with varying bandwidths. By modifying the bandwidth estimation of modes and incorporating fractional-order constraints into the Wiener filter, VFW-VMD demonstrates enhanced adaptability within the VMD framework for decomposing signals with different bandwidths.

Experimental results indicate that VFW-VMD outperforms traditional VMD and other methods in terms of mode separation and fidelity, particularly when processing broadband and nonlinear chirp signals. Moreover, in bearing fault feature extraction, the decomposition results of VFW-VMD retain more fault characteristic information. Additionally, VFW-VMD allows flexible adjustment of the decomposition order *l* based on the signal type. However, the calculation of the envelope spectral entropy of the decomposition results reveals that the adjustment of mode bandwidth constraints inevitably introduces additional noise. This limitation could be addressed in future work through the integration of advanced noise reduction techniques.

Future research may explore incorporating fractional-order derivatives into other mode decomposition techniques and developing adaptive extensions of VFW-VMD to further enhance its performance and applicability.

## Figures and Tables

**Table 1:** $f_{Sig2}\left(t\right)$ Component parameter table.

	$x_{1}\left(t\right)$	$x_{2}\left(t\right)$	$x_{3}\left(t\right)$
*A*	1	1	3
β	200	200	200
*T*	1/220	1/150	1/30
ω	1000	2200	3500

**Table 2:** $f_{Sig3}\left(t\right)$ Error table for VMD and VFW-VMD.

Method	VMD	VFW-VMD
Error of Component 1	0.01949	0.003854
Error of Component 2	0.08275	0.06945

**Table 3:** Vertical centrifugal pump performance parameters.

Type	Flow Rate	Head	Speed	Driver Power	Operating Temperature
GDS50-250	35 m^3^/h	70 m	3950 r/min	18.5 kW	10–60 °C

**Table 4:** Performance parameters of roller bearings.

Type	Basic Rated Dynamic Load	Basic Rated Static Load	Reference Speed	Limiting Speed
NU308 ECM	93 kN	78 kN	8000 r/min	9500 r/min

**Table 5:** The parameters of the vibration signal acquisition system equipment.

Name	Function	Model	Model Specifications	Quantity
Networked intelligent data acquisition device	Data transfer and conversion	INV3062	8-channel/24 bit, with a sampling frequency of 6.25–51.2 kHz per channel, network interface, embedded ARM system, supporting online and offline sampling.	1
Data acquisition and signal analysis systems.	Data shows	DASP-V11	Oscilloscope sampling, INV high-precision frequency meter, time domain analysis, format conversion.	1
IEPE accelerometer sensor	Collect vibration signals	INV9822	M5 installation thread, installation resonance frequency > 25 kHz.	3
Insulated small magnetic seat	Install vibration sensor	INV-CXP6	Small insulated magnetic seat, external M5 thread, suction force 60 N.	3
Shielded cable	Connect sensors and data acquisition devices	Ln-m5Bnc-10m	Shielded cable, one end m5, one end BNC connector, length 10 m.	3

## Data Availability

Data for this article can be obtained by contacting the corresponding author.
